# Supplementary material for: Genetic and maternal environmental contributions to estimated fetal weight at 20 weeks gestation compared with birthweight
Source: medRxiv. 2026 Jun 22:2026.06.18.26355970. Preprint. [Version 1] doi: 10.64898/2026.06.18.26355970 (PMC13320941; doi:10.64898/2026.06.18.26355970)

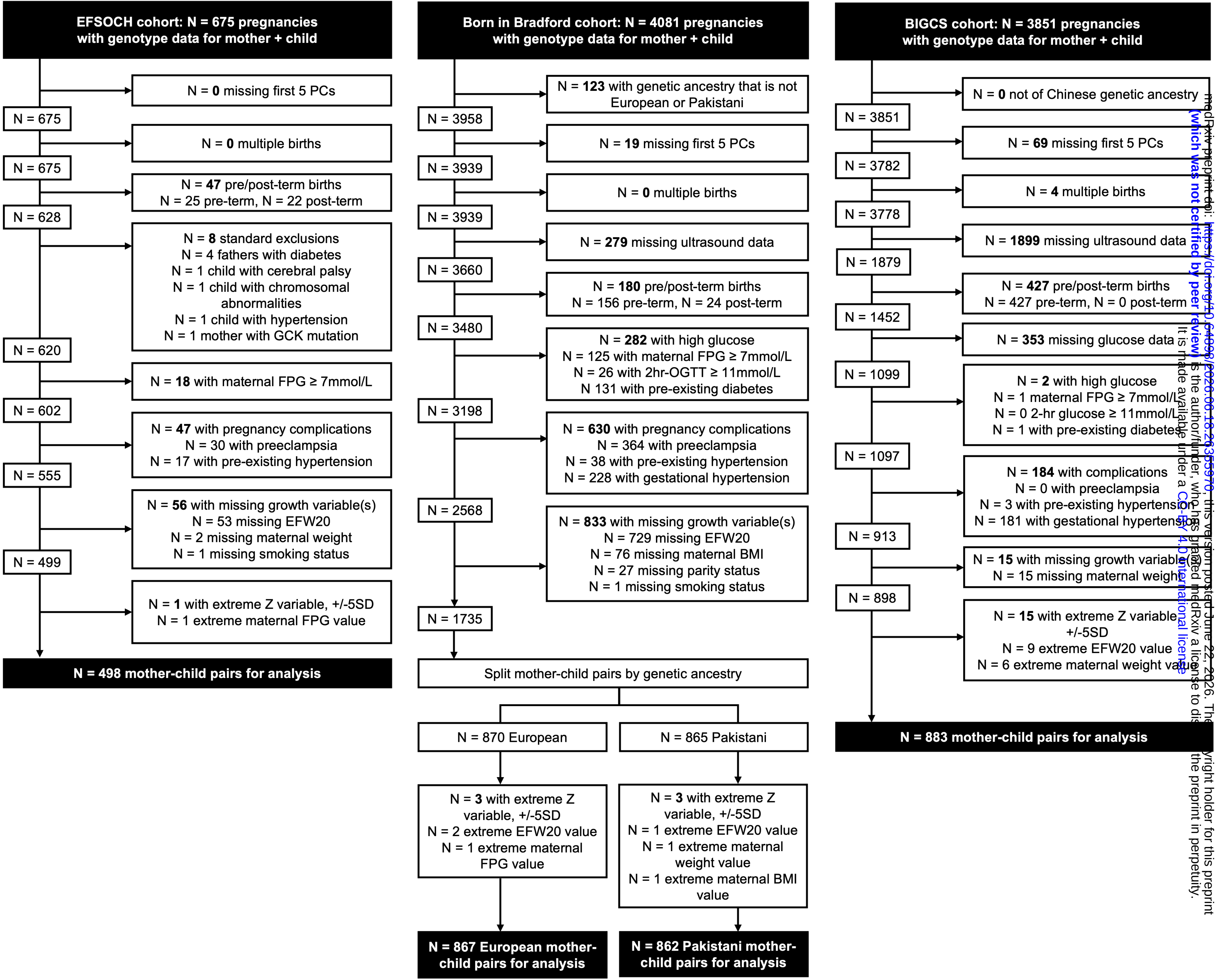

medRxiv preprint doi: https://doi.org/10.64898/2026.06.18.26355970; this version posted June 22, 2026. The copyright holder for this preprint (which was not certified by peer review) is the author/funder, who has granted medRxiv a license to display the preprint in perpetuity.
It is made available under a CC-BY 4.0 International license .

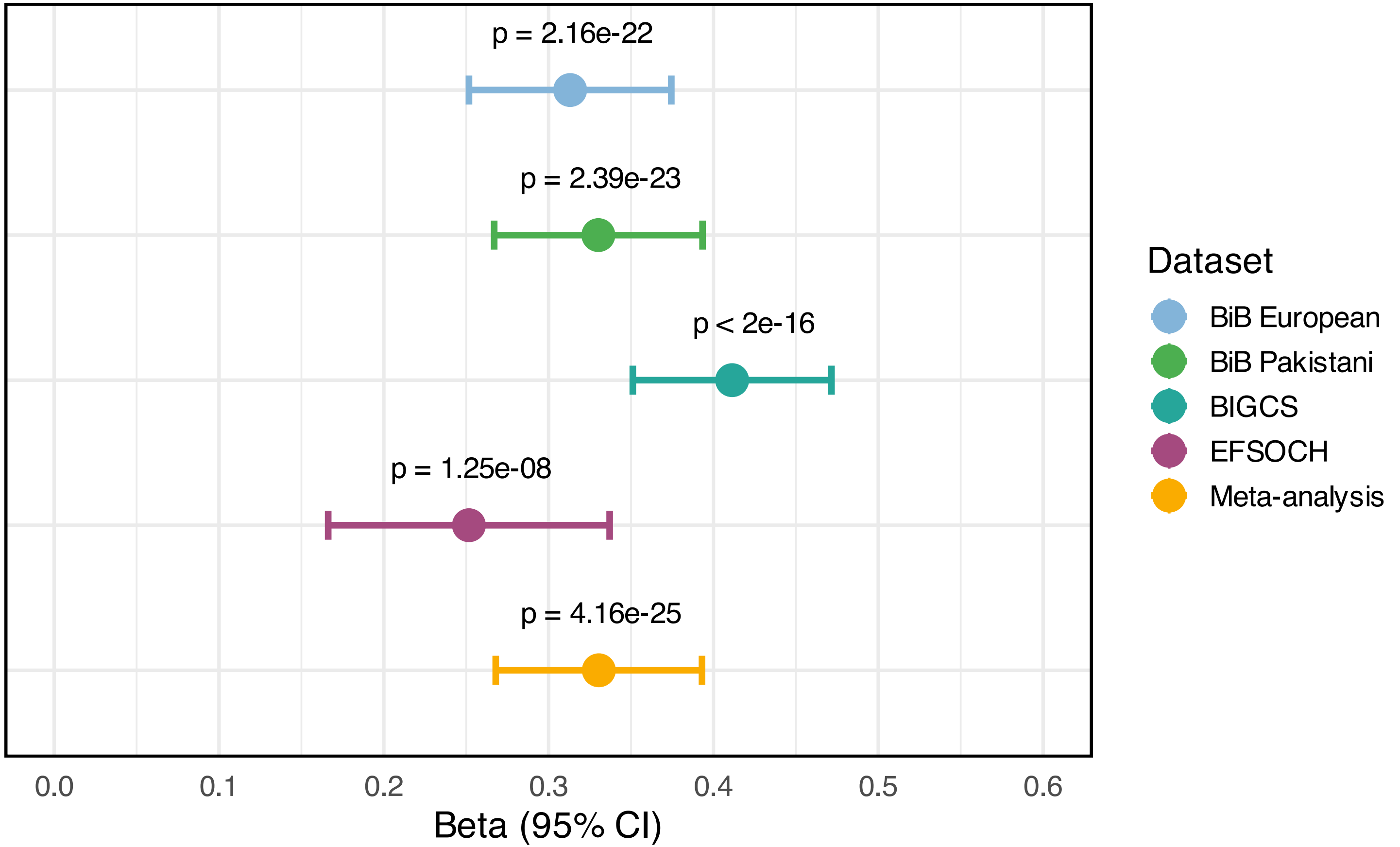

Associations between standardised EFW20 and BW

medRxiv preprint doi: https://doi.org/10.64898/2026.06.18.26355970; this version posted June 22, 2026. The copyright holder for this preprint (which was not certified by peer review) is the author/funder, who has granted medRxiv a license to display the preprint in perpetuity.
It is made available under a CC-BY 4.0 International license .

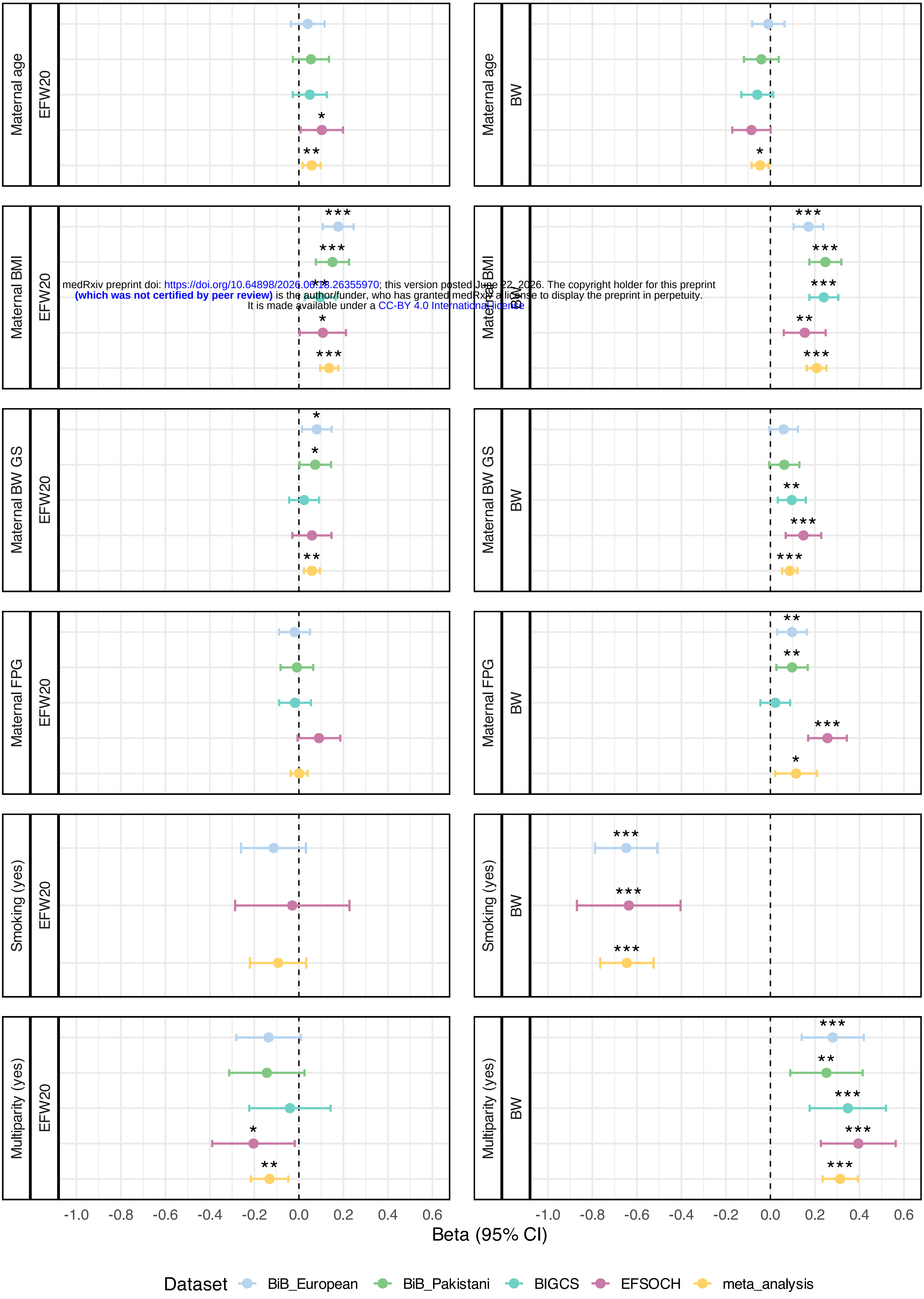

Supplement: 1 — Figure S1. Flowcharts illustrating how the data was filtered and prepared for analysis. EFSOCH (left), BiB (middle) and BIGCS (right). No multiple births were reported when filtering the BiB dataset due to using data from unrelated families, of which only first births were selected. Figure S2. Association between EFW20 (standardized for gestational age at scan) and BW (standardized for gestational age at birth) with p-values for association displayed. Figure S3. Sensitivity analysis of associations between maternal environmental factors and fetal growth outcomes at 20 weeks gestation and at term. Beta value represents standard deviation unit change in grams of estimated fetal weight at 20 weeks (EFW20) or birthweight (BW) per 1 unit change in the standardized or binary exposure variable. * = p <0.05, ** = p <0.01 and *** = p <0.001. Maternal smoking was not included in the BiB Pakistani and BIGCS maternal models due to low sample size of smokers. [file NIHPP2026.06.18.26355970V1-supplement-1.pdf]
